# Dual Antimicrobial Activity of HTCC and Its Nanoparticles: A Synergistic Approach for Antibacterial and Antiviral Applications Through Combined In Silico and In Vitro Studies

**DOI:** 10.3390/polym16212999

**Published:** 2024-10-25

**Authors:** Khanyisile S. Dhlamini, Cyril T. Selepe, Bathabile Ramalapa, Zamani Cele, Kanyane Malatji, Krishna K. Govender, Lesego Tshweu, Suprakas Sinha Ray

**Affiliations:** 1 Centre for Nanostructures and Advanced Materials, DSI-CSIR Nanotechnology Innovation Centre, Council for Scientific and Industrial Research, Pretoria 0001, South Africa; sheer959@gmail.com (K.S.D.); cyrilkatlego15@gmail.com (C.T.S.); bramalapa@csir.co.za (B.R.); zcele@csir.co.za (Z.C.); kmalatji@csir.co.za (K.M.); 2 Department of Chemical Sciences, University of Johannesburg, Doornfontein 2028, Johannesburg, South Africa; krishnag@uj.ac.za; 3 Material Science, Innovation and Modelling (MaSIM), Faculty of Natural and Agricultural Sciences, North-West University, Mmabatho 2735, South Africa

**Keywords:** HTCC, antimicrobial activity, molecular docking, MD simulation

## Abstract

N-(2-hydroxyl) propyl-3-trimethyl ammonium chitosan chloride (HTCC), a quaternized chitosan derivative, has been shown to exhibit a broad spectrum of antimicrobial activity, especially against bacteria and enveloped viruses. Despite this, molecular docking studies showing its atomic-level mechanisms against these microorganisms are scarce. Here, for the first time, we employed molecular docking analyses to investigate the potential antibacterial activity of HTCC against *Staphylococcus aureus* and its antiviral activity against human immunodeficiency virus 1 (HIV-1). According to the findings, HTCC exhibited promising antibacterial activity with high binding affinities; however, it had limited antiviral activity. To validate these theoretical outcomes, experimental studies were conducted. Different derivatives of HTCC were synthesized and characterized using NMR, XRD, FTIR, and DLS. The in vitro assays validated the potent antibacterial efficacy of HTCC against *S. aureus*, whereas the antiviral studies did not show good antiviral activity. However, our research also revealed a promising avenue for further exploration of the antimicrobial activity of HTCC nanoparticles (NPs), since, thus far, no studies have been conducted to show the antiviral activity of HTCC NPs against HIV-1. The nanosized HTCC exhibited superior antiviral performance compared to the parent polymers, with complete (100%) inhibition of HIV-1 viral activity at the highest tested concentration (0.33 mg/mL).

## 1. Introduction

Antimicrobial resistance (AMR) has surfaced as a formidable and urgent threat to public health, presenting substantial obstacles to successfully preventing and managing microbial infections. AMR occurs when microorganisms, like bacteria, viruses, fungi, and parasites, become resistant to the standard drugs used to treat them, making treatment of infections increasingly difficult [1]. A significant study featured in The Lancet in January 2022 reported that approximately 1.27 million fatalities were linked to AMR in 2019 [2]. Furthermore, approximately 5 million deaths were indirectly linked to infections resistant to drugs, emphasizing the severity of the problem. Compounding the issue, a report by Jim O’Neill has projected that the annual death toll associated with AMR could escalate to 10 million by the year 2050 [3]. This rapidly increasing threat of AMR has underscored the critical necessity for identifying innovative antimicrobial materials and developing novel strategies to address this challenge [4,5]. COVID-19 has also contributed to the rise of AMR, as 72% of patients received antibiotics despite only 8% having confirmed bacterial co-infections [6]. This overuse is linked to the fact that secondary bacterial infections often accompany viral infections [7]. As a result, there is a growing need for materials with dual antimicrobial properties to lower the risk of secondary infections. To combat resistant strains, it is essential to develop materials capable of disrupting microbial membranes or inactivating viruses without relying on drugs. By offering alternative methods to control pathogens, these dual-function materials may help mitigate AMR and reduce dependence on conventional antibiotics and antivirals.

Among various biopolymers, chitosan has recently garnered attention as a promising biomaterial due to its biocompatibility, cost-effectiveness, and biodegradability. Chitosan, derived from the deacetylation of chitin, is a linear natural polysaccharide. It is the second most abundant polysaccharide after cellulose [8]. In contrast to cellulose, chitosan possesses inherent antimicrobial properties due to substituting the C-2 hydroxyl group with a primary amine. This primary amino group on the C-2 position of the D-glucosamine repeat unit is protonated into the soluble protonated form NH_3_^+^ at a lower pH (<pKa~6.5) [9,10]. Unlike other Food and Drug Administration (FDA) approved biopolymers, chitosan stands out for its unique cationic characteristics and mucoadhesive properties [11].

However, although chitosan has been established as an attractive biopolymer in the biomedical field, its applications are slightly restricted due to chitosan being water-insoluble at a neutral pH [12]. Water-soluble chitosan derivatives have been developed to overcome this constraint by introducing permanent positive charges into the polymer chains. This modification results in a cationic characteristic that remains soluble independent of the pH of the aqueous medium. Studies involving quaternary chitosan salts have demonstrated enhanced antimicrobial activity against bacteria compared to unmodified chitosan [13,14,15]. This innovative approach expands the potential applications of chitosan in various fields by addressing its water solubility limitations while retaining its desirable cationic and antimicrobial properties.

The most extensively researched quaternary ammonium derivative of chitosan is HTCC. HTCC is favored among chitosan derivatives because of its relatively facile chemical synthesis involving chitosan and glycidyl-trimethyl-ammonium chloride (GTMAC). Numerous studies have explored the antibacterial effectiveness of HTCC against challenging bacterial strains, including *S. aureus* [16,17,18,19,20]. Recent investigations have highlighted HTCC’s potential antiviral properties, particularly against enveloped viruses [16,21,22,23]. However, research on the application of HTCC NPs against such enveloped viruses remains limited. To bridge this gap, HTCC was nanosized with the goal of enhancing its antiviral efficacy, as most studies on HTCC’s antiviral activity show inhibition but often at relatively high concentrations. Furthermore, despite the significant interest in HTCC as an antimicrobial material, there is a noticeable lack of comprehensive reports on the molecular docking and molecular dynamics that explain its antimicrobial mechanisms. Consequently, to fill this void, an integrated experimental and in silico study was conducted in this work, employing a combination of quantum mechanics, molecular docking, and molecular dynamics to elucidate the atomic-level mechanisms of interaction between HTCC and the target proteins.

## 2. Computational Studies

### 2.1. Density Functional Theory (DFT) Studies

DFT is a quantum-mechanical (QM) modeling method used to investigate the electronic structure of molecules [24,25]. The 2D structures of chitosan and HTCC were generated and then converted to 3D structures using ChemDraw Ultra 12.0 [26] and saved as Gaussian input files (.gjf). The 3D structures were transferred to GaussView 6 [27], where they were set up for DFT simulations using M062X/6-311++G (d,p). The molecules were optimized with the aid of Gaussian 16 [28]. The optimized structures were confirmed to be minimal along the potential energy surface by establishing that no negative frequencies were present. The molecular electrostatic potential map (MEP) was generated for each of the optimized molecules considered in this work.

### 2.2. Molecular Docking

The molecular docking technique provides insight into the interaction of the ligands with target proteins at the atomic level and can help us elucidate fundamental biochemical processes. The molecular docking studies were conducted using Schrödinger Maestro 2022-1 [29]. The quantum mechanical optimized structures were imported into Maestro and used for molecular docking. The proteins used for the molecular docking studies were retrieved from the protein databank (PDB): S. *aureus* (PDB ID: 6S7V) and HIV-1 (PDB ID: 4RZ8). The proteins were prepared using the Protein Preparation Workflow, which removed the crystal water molecules and ions, optimized hydrogen bonds, and filled in the missing side chains [30,31].

The protein crystal structure of HIV-1 had a co-crystallized ligand. Therefore, the grid box was generated using the Receptor Grid Generation tool by selecting the co-crystallized ligand inside the protein’s binding site. To check the accuracy of the docking method, the co-crystallized ligands of the target proteins were re-docked into the active site, and the root mean square deviation (RMSD) was calculated between the co-crystallized ligand and the re-docked ligand. The method was considered acceptable if the re-docked ligand showed a low RMSD of less than 2 Å. The RMSD value between the co-crystallized and re-docked ligand was 1.19 Å (Figure S1, Supporting Information) for the HIV-1 protein. However, for the crystal structure of *S. aureus* that did not have a co-crystallized ligand, the sitemap tool was used to predict the potential binding sites and their druggability. The ligand was docked on the site with the highest docking score and druggability (Figure S2, Supporting Information). Molecular docking was conducted using the Ligand Docking tool. For the molecular docking, chitosan and HTCC were considered flexible, while the proteins were considered rigid [30]. The OPLS4 force field was used in all the molecular docking calculations.

### 2.3. Molecular Dynamic (MD) Simulations

The MD simulations of the protein–ligand complexes were performed using the DESMOND (Schrödinger package). The System Builder tool in Maestro was used to prepare a solvent box for the docked complexes. The complexes were placed in a TIP4P orthorhombic water box, and a distance of 10 Å between the protein surface and the boundary of the simulation box was maintained. To neutralize the system, counter ions of Cl^−^ and Na^+^ were added to the system; the buffer concentration was set to 0.15 M to mimic physiological conditions. The MD simulations were carried out in the NPT (constant number of atoms, steady pressure, and continuous temperature) ensemble at 300 K and 1.01325 bar pressure over 200 ns. The OPLS4 force field was used in the simulations [24,32].

## 3. Experimental

### 3.1. Materials

Chitosan (from shrimp shells, ≥75% deacetylated), glycidyl trimethylammonium chloride (GTMAC), sodium tripolyphosphate (TPP), L-(+)-lactic acid (80%), glacial acetic acid (AcOH), silver nitrate (AgNO_3_), potassium chromate, acetone, and Tween-80 were all purchased from Sigma-Aldrich (Modderfontein, South Africa). All other reagents used were of analytical grade. Millipore water was used in all the experiments.

### 3.2. Synthesis of HTCC

The HTCC polymers were synthesized using a previously reported method with slight modifications [18]. Three different derivatives were synthesized under the same conditions; the only factor that was changed was the molar ratio of GTMAC to the sugar unit of chitosan. Briefly, chitosan (1.00 g) was dissolved in an acetic acid solution (250 mL, 0.5% *v*/*v*) and stirred for approximately 6 h to obtain a clear solution. GTMAC was then added to the solution in two portions at 1 h intervals. After adding GTMAC, the solution was stirred for 24 h at 60 °C. The product was precipitated in excess acetone and centrifuged (3000 rpm, 30 min) to remove the acetone. The product was then washed with an acetone–ethanol mixture (1:1) and freeze-dried to give the product as a white foam.

### 3.3. Synthesis of HTCC NPs

HTCC NPs were synthesized using the ionic gelation method previously reported with slight modifications [33]. HTCC (25.0 mg) was dissolved in L-(+)-lactic acid (25.0 mL, 1% *v*/*v*), and then, a few drops of tween-80 were added. TPP solution (12 mL, 0.2% *w*/*v*) was then added to the HTCC solution dropwise until an opalescent suspension was observed, and the solution was lyophilized to give a white foam.

### 3.4. Characterization

The proton nuclear magnetic resonance (^1^HNMR) spectra were recorded on a 600 MHz Varian INOVA. Chemical shifts (δ) were reported in parts per million (ppm) downfield with deuterated water (D_2_O) as a solvent. All the samples were dissolved in D_2_O except for chitosan, which was dissolved in an aqueous solution of deuterated hydrochloric acid (1% DCl/D_2_O *v*/*v*). FTIR analysis was performed using a PerkinElmer spectrum 100 FTIR spectrophotometer (Waltham, MA, USA) between 4000 and 600 cm^−1^. All the spectra were recorded against a background of an air spectrum. X-ray diffraction (XRD) analysis of the polymers was performed using a Phillips X’Pert PRO diffractometer (PANalytical, Almelo, Netherlands), with CuKα radiation (wavelength y = 0.154 nm) operated at a voltage of 45 kV and current of 40 mA within scattered radiation detected in the range of 2 = 5°–90°.

The mean particle size, PDI, and zeta potential of the HTCC and HTTC nanoparticles were assessed by dynamic light scattering using a Malvern Zetasizer Nano ZS (Malvern Instruments, Worcestershire, UK). Measurements were conducted on diluted samples at 25 °C. Each sample was measured three times, and all data were shown as the mean  ±  standard deviation (SD).

Transmission electron microscopy (TEM, Zeiss NTS GmbH, Oberkochen, Germany), operated at an acceleration voltage of 200 kV, observed the surface morphology of the prepared nanoparticles. The samples were prepared by depositing a drop of the HTCC NP solution suspension on a carbon-coated copper grid and drying at room temperature before observation.

### 3.5. The Antibacterial Assays

A serial dilution microplate method [34] was followed to determine the minimum inhibitory concentration (MIC) of the compounds against *Staphylococcus aureus* ATCC 29213. Bacterial cultures were grown overnight in Mueller Hinton broth (Sigma Aldrich, Modderfontein, South Africa) and adjusted to McFarland standard 0.5 with fresh MH media. A total of 100 μL of samples were added to the first well of a sterile 96-well microtiter plate containing 100 μL of sterile distilled water and serially diluted. A total of 100 μL of adjusted bacteria cultures were added to each well. The bacteria were subjected to final compound concentrations of 0.625–0.0098 mg/mL. Gentamicin (Virbac, St. Louis, MO, USA) served as a positive control and was also tested at a final compound concentration of 0.625–0.0098 mg/mL. Broth and water served as sterility controls. The microplates were then incubated at 37 °C for 24 h. After incubation, the plates were removed from the incubator, and 40 μL of 0.2 mg/mL p-iodonitrotetrazolium violet (INT) dissolved in sterile water was added to the wells and incubated further at 37 °C for 1 h. The MIC was determined visually as the lowest concentration that led to growth inhibition [34]. The assay was performed in triplicate and repeated twice.

### 3.6. Antiviral Activity Against Pseudo-HIV Virus

#### 3.6.1. Cytotoxicity Assay

The cytotoxicity assay for the HTCC samples was performed by first seeding 10k cells/well/100 µL of TZM-bl cells in a 96-well plate for 24 h at 37 °C and 5% CO_2_. After 24 h, a 3-fold serial dilution of the samples was performed using a starting concentration of 0.333 mg/mL. To the plate containing cells, 100 µL of the samples’ serial dilutions was added, except in the cell control wells. The cells were then incubated with the samples for 48 h. Following the 48 h incubation, the media was removed and replaced with 25 µL of 5 mg/mL 3-(4,5-Dimethylthiazol-2-yl)-2,5-diphenyltetrazolium bromide (MTT) reagent (Thermo fisher scientific, Waltham, MA, USA). This was followed by 3 h incubation at 37 °C to allow for the formazan product to form from viable cells. After incubation, the MTT was removed, and 100 µL dimethyl sulfoxide (DMSO) was added and incubated for 15 min at room temperature. Afterwards, the plates were read at a wavelength of 620 nm using the Tecan Infinite F500 luminometer (Zürich, Switzerland).

#### 3.6.2. Generation of HIV-1 Env-Pseudoviruses

The method followed was outlined by London et al. [35]. Briefly, the HIV-1 CAP210.2.00.E8 subtype C pseudovirus was generated by co-transfection of the envelope containing plasmid (PSG-3Δenv), with a plasmid carrying the luciferase reporter gene [36] into 2 × 10^6^ HEK 293T cells/10 mL of growth media using the X-tremeGENE transfection reagent (Sigma Aldrich, St. Louis, MO, USA). The 50% tissue culture infectious dose (TCID50) of the virus stock was quantified by infecting TZM-bl cells with serial 4-fold dilutions of the supernatant in quadruplicate in the presence of DEAE dextran (37.5 μg/mL) (Sigma-Aldrich, St. Louis, MO, USA). The Bright Glo™ Reagent (Promega, Madison, WI, USA) was used to measure infection after 48 h of tissue culture, according to the manufacturer’s instructions. Luminescence was measured using a Tecan Infinite F500 (Zürich, Switzerland).

#### 3.6.3. Assay Set-Up to Investigate the Inhibition of HIV-1 Subtype C Infection of TZM-bl Cells

The experiment was performed in duplicates in a 96-well flat-bottom plate. To all wells except the cell control wells, 100 µL of Dulbecco’s Modified Eagle Medium (DMEM) growth media supplemented with fetal bovine serum (FBS) (Thermo Fisher Scientific, Waltham, MA, USA) was added. In comparison, 150 µL was added to the cell control wells. A 3-fold serial dilution of the HTCC samples was performed from the starting concentration of 0.333 mg/mL. After dilution of the samples, 50 µL of CAP210.2.00.E8 pseudovirus was added and incubated for 1 h at 37 °C to allow for the samples to interact with the pseudovirus. Virus control wells were also included (no HTCC samples). This was followed by the addition of 10k cells/well/100 µL of TZM-bl cells and incubation for 48 h at 37 °C and 5% CO_2_. After the 48 h incubation, 150 µL of media was removed from the plates, followed by adding 100 µL Bright-Glo Luciferase substrate (Promega, Madison, WI, USA) and incubation for 2 min. After that, 150 µL was transferred to 96-well black plates, and luminescence was read using a Tecan-i-control, Infinite F500 (Zürich, Switzerland), and infection was recorded as Relative Light Unit (RLU).

## 4. Results and Discussion

### 4.1. Density Functional Theory

#### 4.1.1. Structure Optimization

**Figure 1:**
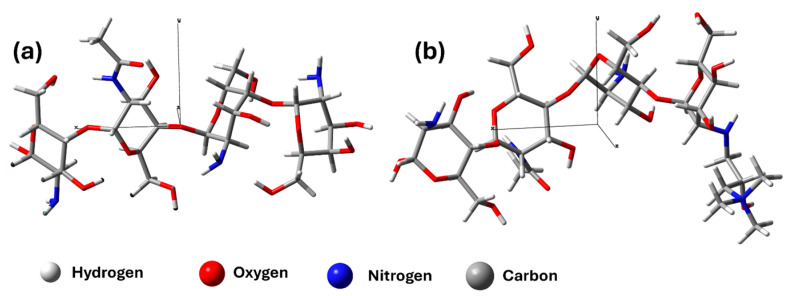
The optimized geometric structures of (**a**) chitosan and (**b**) HTCC-1 were calculated using M062X/6-311++G (d,p).

The structures were optimized using geometry optimization to obtain the most stable geometry of the polymers. Although chitosan is typically a longer polymer, only four chains were modeled in this study for computational convenience. The chitosan used in the experimental work had a 75% DA. To replicate this, one chain was modeled as an N-acetyl glucosamine unit, while the other three chains represented glucosamine units. For the molecular docking studies, the effect of the DQ was investigated by substituting the hydrogen atoms on the amino groups of the glucosamine units in chitosan with GTMAC, resulting in 33% (HTCC-1) DQ. Figure 1 illustrates the most stable geometries of chitosan and HTCC. These optimized structures were used to ensure minimum energy conformations for the molecular docking analysis.

#### 4.1.2. Molecular Electrostatic Potential Map

**Figure 2:**
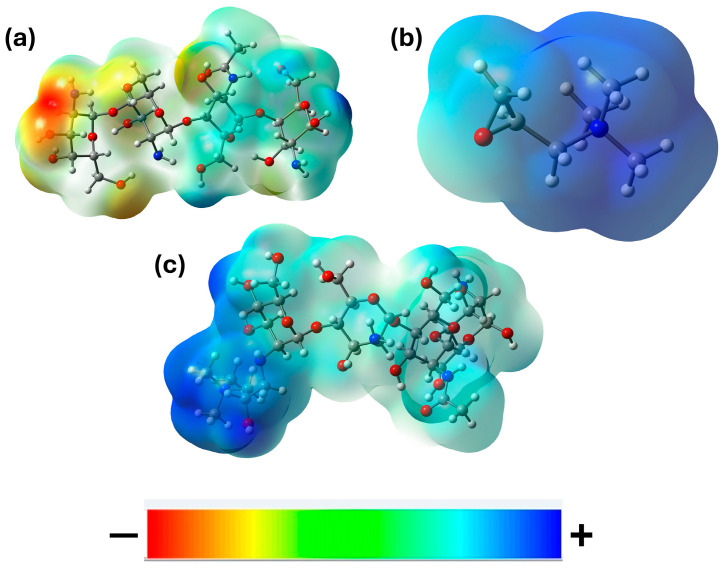
Molecular electrostatic potential map of (**a**) chitosan, (**b**) GTMAC, and (**c**) HTCC-1.

The MEP provides a visual representation of the charge distribution in a molecule, showing possible sites that can undergo electrophilic or nucleophilic attack [37]. As shown in Figure 2, the color scheme determines the electrostatic potential for MEP maps. Red corresponds to regions of high electron density (the most negative region), blue corresponds to regions of low electron density (the most positive region), and yellow and green correspond to intermediate levels [38]. In Figure 2a, due to the oxygen and nitrogen atoms in the hydroxyl and amine groups, these groups concentrate on a high density of negative charges. As a result, these groups are more susceptible to electrophilic attack. In Figure 2b, the entire molecule displays a blue to light blue color, indicating a high positive charge density, which is expected given the positive charge of GTMAC. Similarly, in Figure 2c, after conjugation with GTMAC, the chitosan molecule also shows a blue to light blue coloration, reflecting the permanent positive charge introduced by the quaternary ammonium salt group on the chitosan backbone.

### 4.2. Molecular Mechanics

#### Molecular Docking Studies

**Figure 3:**
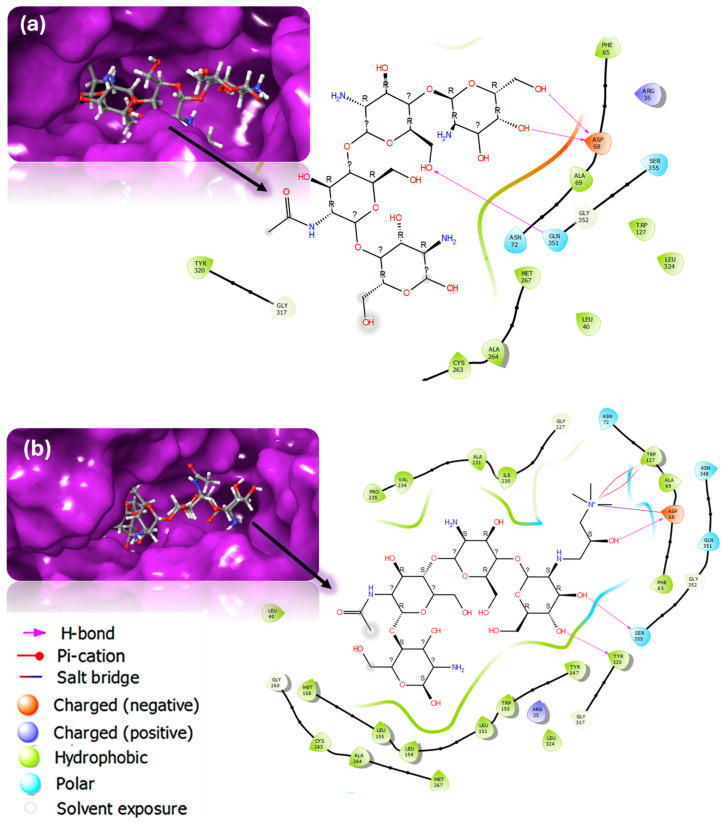
The 2D and 3D images of the ligands docked inside the *S. aureus* Lta pocket and their interactions: (**a**) Chitosan and (**b**) HTCC-1.

The molecular docking approach can be used to model the interaction between molecules and proteins at the atomic level [39]. Molecular docking studies were conducted to provide insights into the specific interactions between the polymers and the protein structures of *S. aureus* and HIV-1. The docking score of the ligand into the protein’s binding pocket is thought to represent the sum of the observed interactions. The more negative the value for the docking score, the more the ligand has a binding affinity towards the target protein. Previous studies showed that chitosan’s antibacterial activity and its derivatives differ from antibiotics, which generally inhibit specific cellular processes. In this work, we used crystal structures of membrane proteins for molecular docking since chitosan and its derivatives target the bacterial cell membrane, according to the literature. The crystal structure of lipoteichoic acid (LTA) (PDB ID: 6S7V) was used for the molecular docking of *S. aureus*. LTA is a major constituent of the cell wall of Gram-positive bacteria. A key role of LTAs is the maintenance of cell wall structure, regulation of autolytic enzymes, and interaction with the host’s immune system [40]. The docking scores of the *S. aureus* LTA protein for chitosan and HTCC-1 were −5.72 and −6.50 kcal/mol, respectively. Chitosan formed hydrogen bonds with the Asp68 and Gln351 amino acids (Figure 3a). Compared to chitosan, HTCC-1 displayed a higher binding affinity due to pi-cation interactions with Trp127, salt bridge formations with Arg68, and hydrogen bonds with Arg68 from the GTMAC functional groups (Figure 3b). Additional hydrogen bonds were formed between the chitosan backbone and Tyr320 and Ser355. The molecular docking analysis indicates that HTCC has a strong binding affinity toward the *S. aureus* protein, suggesting its potential to inhibit *S. aureus* growth.

**Figure 4:**
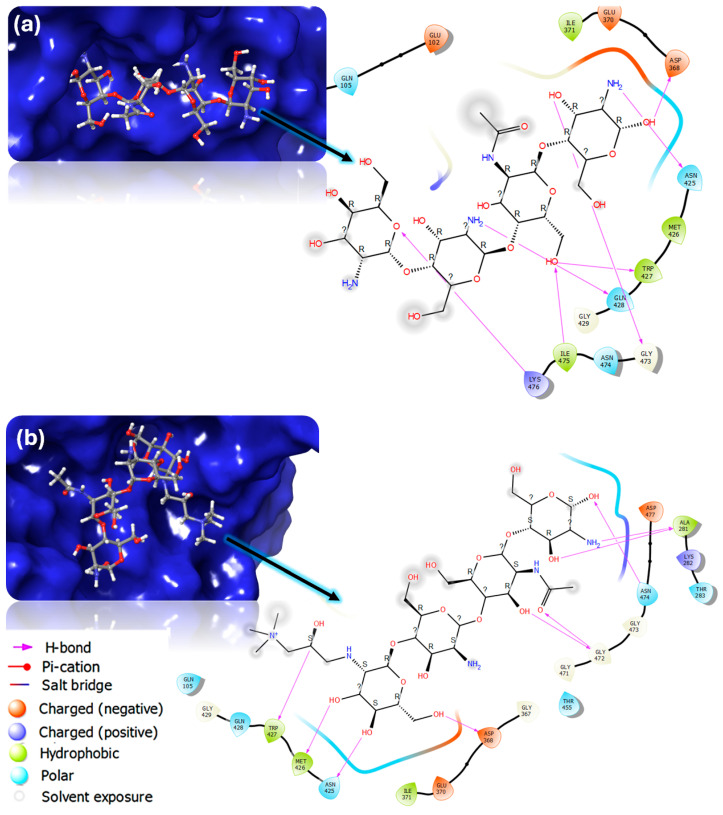
The 2D and 3D images of the ligands docked inside the HIV-1 gp120 protein pocket and their interactions: (**a**) Chitosan and (**b**) HTCC-1.

Molecular docking was also employed to assess the antiviral activity of the polymers against the HIV-1 viral protein gp120 core (PBD ID: 4RZ8). The gp120 protein is crucial for recognizing and entering the target cell by binding to CD4 receptors on the host cell surface, making it a primary target for entry inhibitors [41]. The docking scores for chitosan and HTCC-1 against the HIV-1 gp120 protein were −4.21 and −5.08, respectively. Figure 4a shows that chitosan formed hydrogen bonds with the Asp368, Asn425, Trp427, Gln428, Gly473, Ile475, and Lys476 amino acid residues of HIV-1 gp120 protein. In comparison, HTCC-1 formed hydrogen bonds with the Ala281, Asp368, Asn425, Met426, Trp427, Gly472, and Asn474 residues (Figure 4b). The molecular docking results between the polymers and HIV-1 gp120 protein showed that they interacted by forming hydrogen bonds. The binding affinity increased after modifying chitosan, which correlates with many experimental studies that have shown that HTCC has a higher antiviral activity compared to chitosan. However, based on the molecular docking results, the enhanced antiviral activity against HIV-1 is attributed to the hydrogen bonding between the hydroxy groups of GTMAC and the amino acids of the gp120 protein. A higher degree of substitution (DS) leads to increased activity due to more interactions between the protein’s amino acids and the hydroxy groups of GTMAC. Another key observation from the docking studies is that HTCC-1 (Figure 4b) interacts with three hydrophobic amino acids: Ala281, Met426, and Trp427. Since hydrophobic interactions are generally weaker and less specific than hydrogen bonding and ionic interactions, HTCC-1 may exhibit reduced binding affinity to the target protein when it interacts more with hydrophobic amino acids, leading to less specific and weaker binding. The polymers exhibited a low binding affinity towards the HIV-1 protein gp120. Thus, it is likely that HTCC will not have good antiviral activity against HIV-1.

### 4.3. Molecular Dynamics Simulation

**Figure 5:**
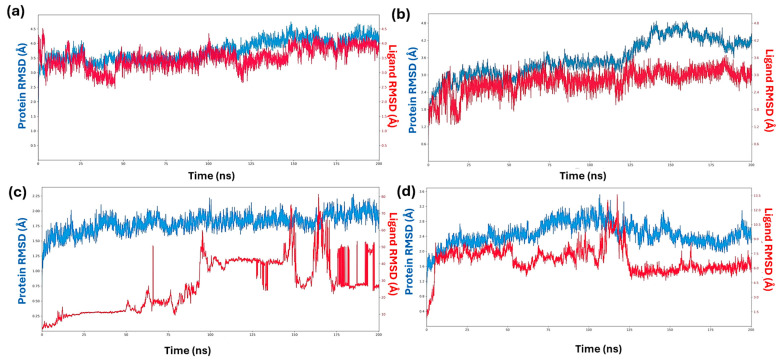
The RMSD plot of MD simulation trajectory obtained after 200 ns for (**a**) Chitosan–*S. aureus* complex, (**b**) HTCC–*S. aureus* complex, (**c**) Chitosan–HIV-1 complex, and (**d**) HTCC–HIV-1 complex.

The MD simulations were conducted for the chitosan and HTCC-1 complexes formed with HIV-1 and *S. aureus* proteins to better understand the overall stability of the complexes after a specific time (in nanosecond timescale). The complexes were subjected to 200 ns MD simulations, and the root mean square deviation (RMSD) was used to check the stability of the complexes. The RMSD of the proteins gives insights into the protein’s structural confirmation during the simulation. Figure 5a presents the RMSD plot of chitosan with *S. aureus* LTA. The protein and ligand begin to stabilize at approximately 50 ns, reaching an RMSD value of 3.5 Å, indicating that both the protein and ligand remain stable, with the ligand intact at its initial binding site. In contrast, Figure 5b shows the RMSD plot for HTCC-1 with S. aureus LTA, where the protein stabilizes between 61 ns and 125 ns with an RMSD of 3.3 Å but fluctuates afterward, eventually stabilizing again with an RMSD value of 4.1 Å. The ligand stabilizes earlier, at approximately 25 ns, with an RMSD of 3.1 Å throughout. The small difference in RMSD values suggests that the ligand remains in its initial binding site. Figure 5c,d illustrates the RMSD plots of chitosan and HTCC-1 with the HIV-1 gp120 protein. The RMSD plot for chitosan shows significant fluctuations in the ligand with no stabilization, suggesting the ligand has diffused away from its initial binding site (Figure S3). This was expected, given the low binding affinity of chitosan for the HIV-1 protein. In contrast, for HTCC-1, the ligand stabilizes at approximately 125 ns with an RMSD of 6.1 Å.

### 4.4. Synthesis and Characterization of HTCC Derivatives

**Scheme 1:**
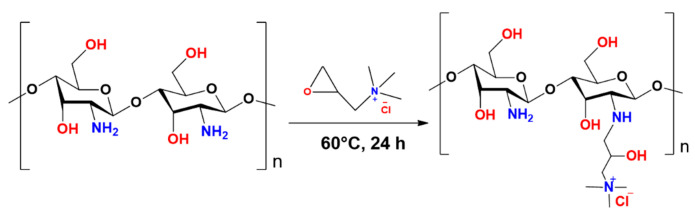
Synthesis of cationic chitosan derivative HTCCs by reacting chitosan with GTMAC.

The cationic chitosan derivatives: HTCCs were synthesized by reacting the amino groups of chitosan with GTMAC, as illustrated in Scheme 1. Three different molar ratios of GTMAC to amino groups of chitosan, specifically 8:1, 6:1, and 4:1, were prepared. The chitosan’s molar ratio, molecular weight, and degree of deacetylation remained constant throughout the process. Under these reaction conditions, the preferential grafting took place specifically on the primary amine groups of chitosan, as amines demonstrate higher nucleophilicity than hydroxyl groups.

The DQ was determined by conductometric titration of Cl^−^ ions with AgNO_3_ solution. Table 1 shows the obtained DQ for the three different HTCC derivatives; the DQs for HTCC-1, HTCC-2, and HTCC-3 were determined to be 31, 47, and 58%, respectively. The zeta potentials for HTCC-1, HTCC-2, and HTCC-3 were 22, 33, and 59 mV, respectively (Figure S3). The maximum solubilities for materials HTCC-2 and HTCC-3 were determined to be between 35 and 40 mg/mL. However, HTCC-1 would form a gel at concentrations as low as 10 mg/mL. An increase in the DQ increased surface charge and solubility.

**Figure 6:**
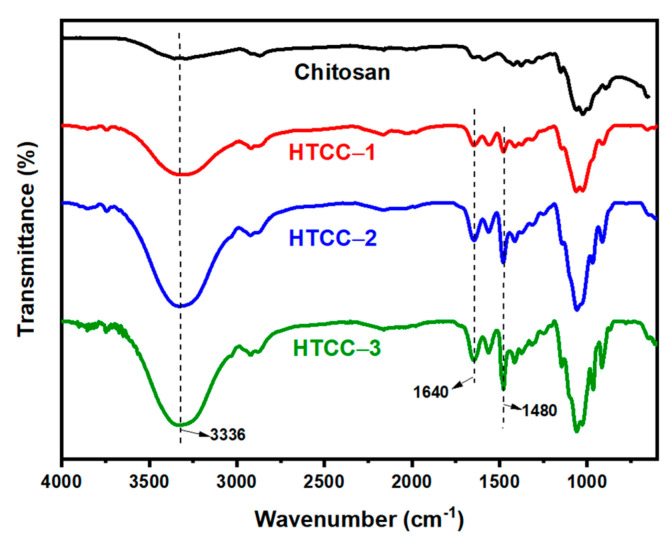
FTIR spectra of chitosan and HTCC derivatives.

The ATR-FTIR spectra of chitosan and the HTCC derivatives are shown in Figure 6. The chitosan spectrum exhibited notable features: a prominent peak at 3336 cm^−1^, corresponding to the O-H stretching vibration, and another at 3285 cm^−1^, indicative of N-H stretching. Additionally, absorption bands at 2906 and 2869 cm^−1^ are attributed to C-H symmetric and asymmetric stretching, respectively. The band at 1643 cm^−1^ signifies the presence of residual N-acetyl groups, denoting the C=O stretching of amide I. The band at 1570 cm^−1^ is associated with the N-H bending vibration of the primary amine.

Furthermore, the 1416 and 1372 cm^−1^ absorption bands correspond to CH_2_ bending and CH_3_ symmetrical deformations, respectively [42,43,44,45]. In contrast, the HTCC spectrum revealed two distinctive bands at 1480 and 1640 cm^−1^, signifying the successful synthesis of HTCC. The band at 1480 cm^−1^ is attributed to the CH_3_ bending of trimethylammonium, indicating the introduction of quaternary ammonium salt groups onto the chitosan backbones. The band at 1640 cm^−1^ suggests the formation of secondary amines, indicating the conversion of primary amines to secondary amines due to reactions at NH_2_ sites on the chitosan backbones. Notably, the primary amine band at 1585 cm^−1^ disappears, underscoring that the epoxide in GTMAC primarily reacted with the amino groups rather than the hydroxy groups. Finally, the band at 3336 cm^−1^ exhibited broadening, reflecting increased hydroxyl groups. In agreement with previous reports, the FTIR results confirmed the successful formation of HTCC derivatives [46,47].

**Figure 7:**
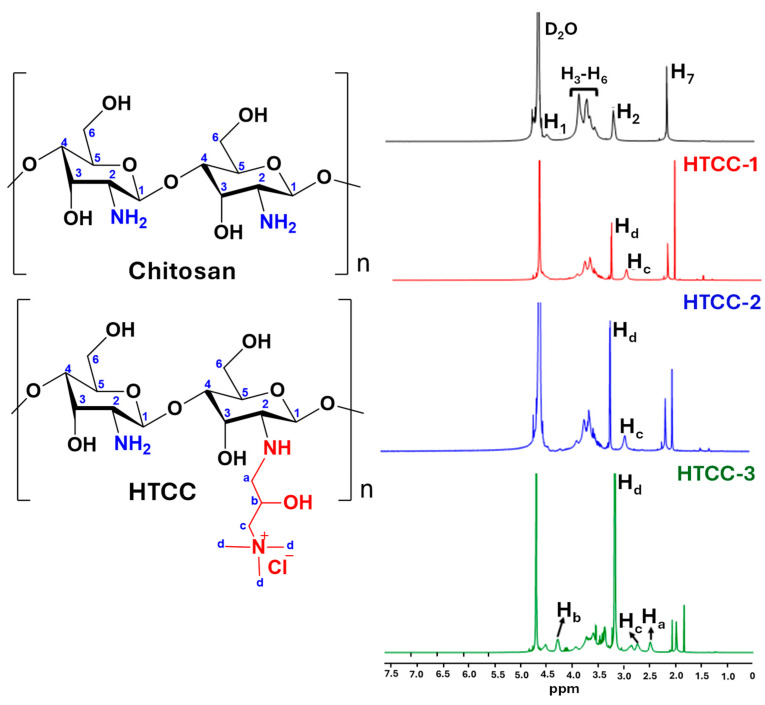
^1^H NMR spectra of chitosan and HTCC derivatives in D_2_O.

The introduction of the quaternary ammonium groups onto chitosan was further confirmed with ^1^H NMR, as presented in Figure 7. The chemical shift at 2.0 ppm is assigned to the acetyl groups’ methyl protons (H7). The chemical shift at 3.18 ppm is assigned to the glucosamine residues proton (H_2_). The chemical shift at 3.6–3.89 ppm is assigned to the non-anomeric protons of chitosan (H_3_, H_4_, H_5_, and H_6_). The chemical shift at 4.5 ppm is ascribed to anomeric protons of chitosan (H_1_) [43,44,45,46]. A new characteristic chemical shift is observed at 3.2 ppm, assigned to the trimethylammonium protons (H_d_), suggesting the successful grafting of GTMAC onto the chitosan backbone. New chemical shifts are also observed at 2.45 and 2.7 ppm, assigned to the methylene group protons (H_a_ and H_c_), and at 4.3 ppm, assigned to the methine group protons (H_b_) on the side chain of HTCC. The GTMAC protons H_a_ and H_b_ could not be observed in HTCC-1 and HTCC-2; however, with HTCC-3, they start becoming more visible. It can also be observed that, the higher the DS, the more prominent the H_d_ protons become. These results were consistent with those in the literature [14,48,49,50].

### 4.5. Physicochemical Properties of HTCC NPs

**Figure 8:**
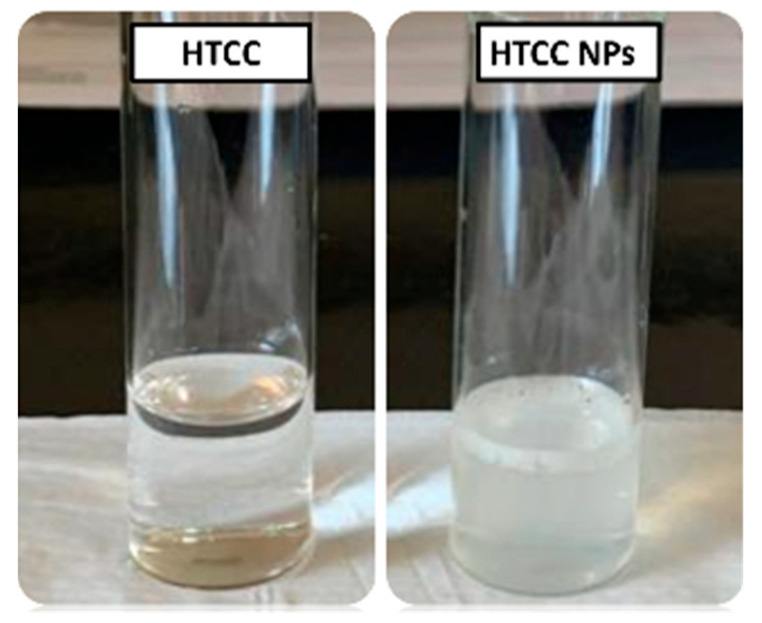
The formation of HTCC NPs through ionic crosslinking.

HTCC NPs were synthesized using the ionic gelation method, and TPP was used as the crosslinking agent. In the ionic gelation method, HTCC NPs are prepared by the ionic interaction between the positively charged amino groups of HTCC and the negatively charged TPP counterions (P_3_) [51]. The pH of TPP was adjusted to 3 to facilitate crosslinking between the phosphoric ions and NH_3_^+^ groups of HTCC. The pH was adjusted to 3 so that only phosphoric ions could be present because, when the pH is high, both the OH^−^ and phosphoric ions will be present and, thus, may compete to interact with the NH_3_^+^ groups [52]. The pH of the HTCC solution was below 5 because the NH_2_ groups of HTCC are mostly protonated and more accessible to interact with. The appearance of the opalescence solution indicated the formation of NPs (Figure 8).

**Figure 9:**
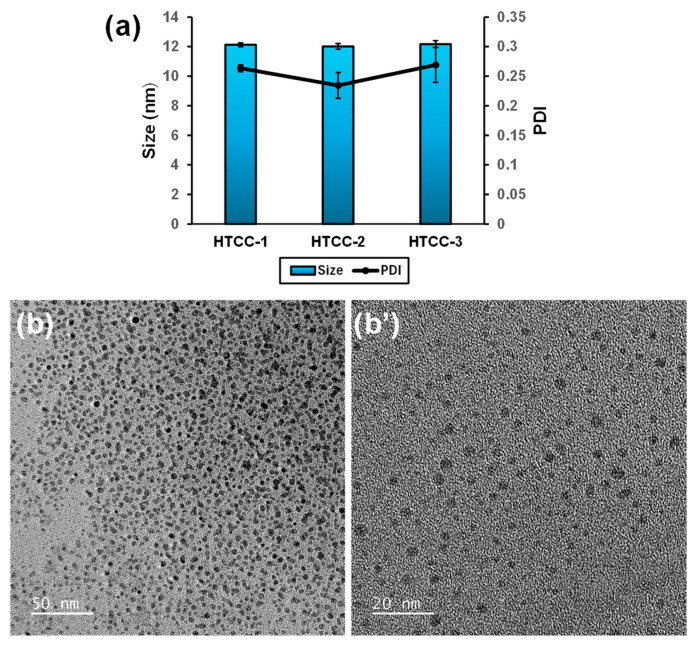
HTCC NPs’ (**a**) size and PDI obtained with DLS (**b**,**b′**) TEM images. HTCC-3 NPs were used for TEM analysis because they were more stable and supported by a high zeta potential value (Table 1).

Under the optimized conditions, the mean particle size of the HTCC NPs was less than 15 nm, with a PDI of less than 0.3, indicating the uniformity of the NPs (Figure 9a). The zeta potential of the HTCC NPs decreased compared to the parent polymer. Rodolfo and his colleagues [53] also noted a similar pattern in their research, revealing that TPP might reduce zeta potential values. This effect is attributed to the increased overall negative charge of the solution due to the addition of TPP [53]. The zeta potential indicates the surface charge of the NPs in the solution. A higher surface charge is desirable to deter agglomeration or aggregation. The morphology of one of the lyophilized HTCC NPs was analyzed using TEM, as shown in Figure 9b. The TEM photomicrograph showed that the NPs were predominantly spherical; however, compared to the results obtained with DLS, the particle sizes were a bit smaller. DLS measurements can be greatly affected by particle aggregation. Typically, DLS measures the average size of particles in a population (suspension); larger aggregates scatter more light, disproportionately influencing the results towards a larger average particle size, as opposed to TEM, which visualizes individual particles, allowing for direct observation of particle aggregates [54].

**Figure 10:**
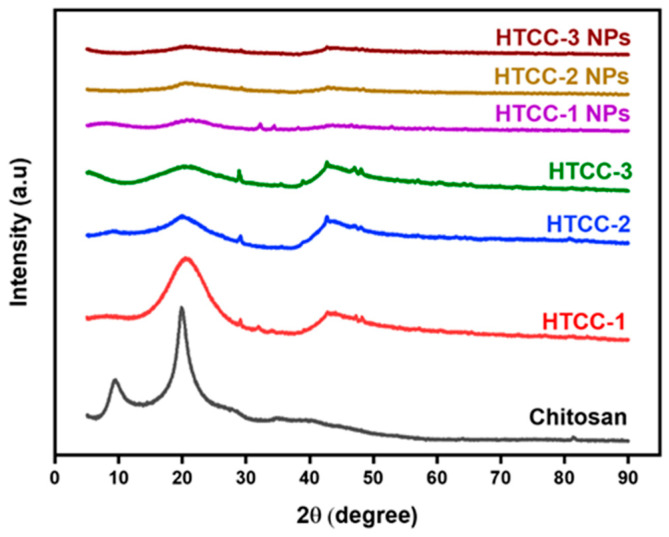
The X-ray diffraction patterns of chitosan, HTCC derivatives, and HTCC NPs.

X-ray diffraction analysis was employed to assess the crystalline patterns of chitosan, HTCC derivatives, and HTCC NPs, as depicted in Figure 10. Chitosan displayed distinct peaks at 2θ = 10° and 2θ = 20°, signifying its semi-crystalline nature. This semi-crystallinity can be attributed to intra-molecular hydrogen bonds formed by amino groups at the C-2 and hydroxyl groups at the C-3 and C-6 positions [50]. In contrast, the XRD patterns of HTCC revealed a peak at 2θ = 20°, albeit with broader characteristics. Notably, the discernible peak at 2θ = 10° disappears gradually, indicating a substantial alteration in the crystal structure of chitosan due to the grafting of quaternary ammonium groups onto its molecular chains. This modification disrupted the pre-existing hydrogen bonds as the bulkier quaternary ammonium groups replaced them. As a result, this interference with the regular crystal lattice arrangement increased the water solubility of the HTCC polymers. The increased solubility transitioned towards an amorphous state, reducing the overall crystallinity [55]. These results were consistent with those in the literature [47,50]. The XRD patterns of the different ratios of HTCC became broader when the DQ increased. Furthermore, the broadening of the peak observed in the X-ray diffraction patterns of the HTCC NPs indicated even more excellent solubility of HTCC in its nanoform.

### 4.6. Antibacterial Activity: Disk-Agar Diffusion and Minimum Inhibitory Assay

The antimicrobial efficacy of HTCC and its NP counterpart was assessed against *S. aureus*, a Gram-positive bacterium. The disk-agar diffusion method was employed to evaluate the antibacterial activity of the samples against the bacterial strains. The experiments were conducted in triplicate, with all samples tested at a concentration of 1 mg/mL. The results indicated that the polymers tested demonstrated antibacterial activity against *S. aureus*, as evidenced by the inhibition zones (Figure S5). The diameter of the inhibition zones for each sample against the bacterial strains is detailed in Table 2.

Although the disk-agar diffusion method revealed antibacterial activity, it can only provide qualitative information. To obtain more quantitative data, the broth microdilution method was used to determine the MIC of the polymers. The MIC is the minimum concentration needed for the compounds to inhibit the growth of bacteria [56]. According to the MIC results (Table 3), HTCC-3 and HTCC-2 displayed the highest antibacterial activity against *S. aureus*, with a MIC value of 156 μg/mL. In comparison, HTCC-1 exhibited the lowest activity, with a MIC value of 313 μg/mL. The results obtained were consistent with previous studies. Kerwald et al. [16] studied the antibacterial activity of different HTCC derivatives with MIC values ranging from 156 to 312 μg/mL. Another study by Hoque et al. [17] also obtained similar results, with MIC values ranging from 125 to 250 μg/mL. The results obtained indicate that increasing the DQ leads to an increase in the antibacterial activity. This is because introducing the positively charged quaternary ammonium salt group N(CH_3_)_3_^+^ endows the surface of HTCC with a large quantity of positive charges [57]. Since the lipoteichoic acids (LTAs) negatively charge the bacterial cell wall, the positively charged HTCC interacts with it to alter cell permeability through electrostatic interactions [58]. This results in the breakdown of the membrane potential, causing leakage of cellular components and, eventually, the death of the bacterial cell [57,59]. HTCC NPs showed increased antibacterial activity; this was indicated by the decrease in the MIC values, with HTCC-1 NPs, HTCC-2 NPs, and HTCC-3 NPs having MIC values of 156, 78, and 78 μg/mL, respectively, against *S. aureus*. The small size increases the ability of NPs to penetrate most physiological barriers and reach their intended targets. The high surface-to-volume ratio increases their capacity to interact with pathogen membranes and cell walls. Similar results were obtained by Lu et al. [60] where, after nanosizing HTCC, the antibacterial activity against *S. aureus* increased significantly.

### 4.7. Antiviral Activity

#### 4.7.1. Cytotoxicity Evaluation on TZM-bl Cells

**Figure 11:**
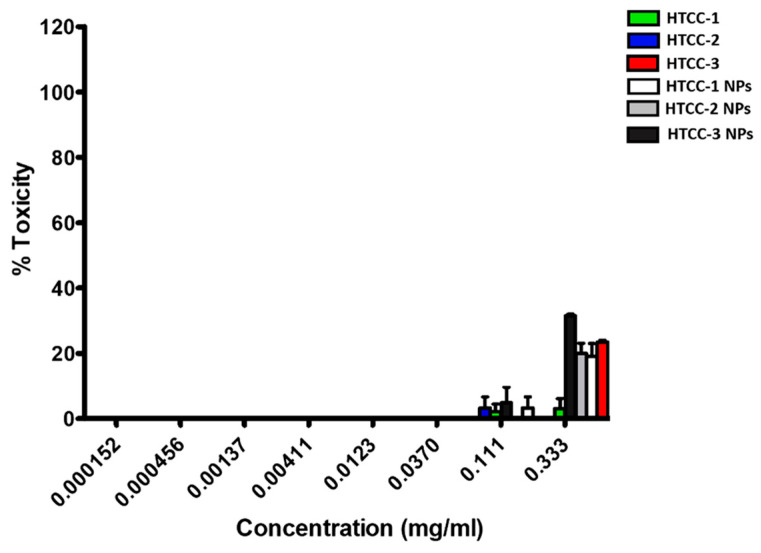
The evaluation of the cationic HTCC derivatives’ and HTCC NPs’ cytotoxicity against TZM-bl-cells. The data shown represent the mean of three independent experiments, and error bars represent the mean ± standard deviation.

The cytotoxicity of the HTCC derivatives and their NPs were evaluated against TZM-bl-cells, as shown in Figure 11. HTCC-1, HTCC-2, and HTCC-3 cytotoxicity were 5, 2, and 20% at the highest tested concentration of 0.333 mg/mL. As shown by the cytotoxicity results, all the synthesized HTCC derivatives were not toxic to the TZM-bl-cells, since the results were below 50%. The cytotoxicity assays were also performed for the NPs, with HTCC-1 NPs, HTCC-2 NPs, and HTCC-3 NPs having cytotoxicity values of 18, 19, and 30%, respectively. The NPs were also not toxic to the TZM-bl-cells. The toxic concentration that kills 50% of the target cells (TC50) could not be determined because, at all tested concentrations, the samples showed toxicity of less than 50% (Figure 10). Thus, the TC50 value exceeds the highest tested concentration of 0.333 mg/mL.

#### 4.7.2. Antiviral Activity Against HIV-1 Subtype C Pseudovirus

**Figure 12:**
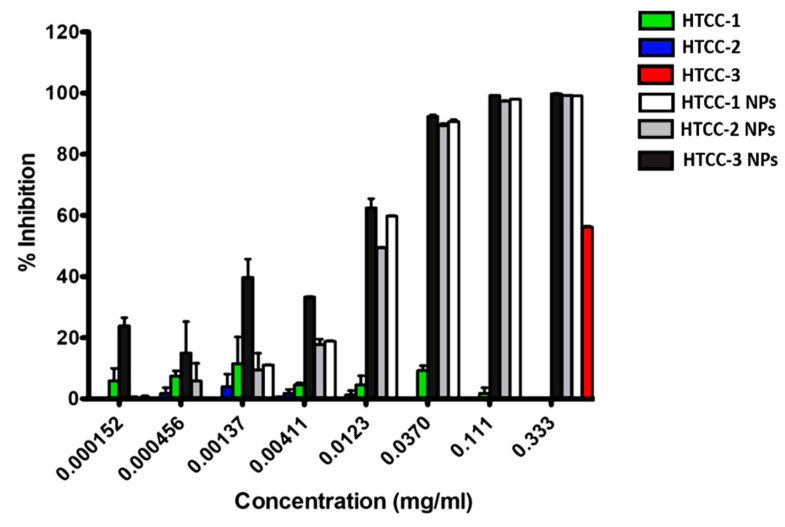
Evaluation of the cationic HTCC derivatives’ and HTCC NPs’ inhibition against HIV-1 subtype C pseudovirus. The data shown represent the mean of three independent experiments, and error bars represent the mean ± standard deviation.

The HTCC derivatives and HTCC NPs were also tested against HIV-1 CAP210.2.00.E8 subtype C pseudovirus. The concentrations used in the inhibition studies were informed by the toxicity study performed on the samples; only the non-toxic concentrations were used for the inhibition studies. Figure 12 shows the antiviral efficacy of the HTCC derivatives and HTCC NPs evaluated using the luciferase-based antiviral assay against HIV-1 subtype C pseudovirus. At the highest tested concentration of 0.333 mg/mL, HTCC-3 showed 58% inhibition of HIV-1. However, other HTCC derivatives (HTCC-1 and HTCC-2) exhibited less than 30% inhibition across various tested concentrations. It is worth noting that, despite having identical chemical structures, all the synthesized HTCC derivatives displayed varying degrees of anti-HIV-1 inhibition, likely attributed to differences in quaternization levels. Most studies on the antiviral activity of HTCC have focused on SARS-CoV-2 and MERS-CoV, where the primary mechanism of inhibition is blocking the interaction between the spike (S) protein and the cellular receptor, thus preventing viral entry into host cells [23,61]. However, a recent study by Cele et al. [21], the only study to date on HTCC’s antiviral activity against HIV-1, reported weak inhibition, achieving 100% inhibition only at a concentration of 280 mg/mL. These findings align with the results of this study, where even at the highest tested concentration, HTCC demonstrated low inhibition activity. This suggests that HTCC is more effective against coronaviruses than against HIV-1, despite some similarities between the two viruses. Studies on chitosan sulfate derivatives against HIV-1 have shown strong inhibition even at low concentrations, attributed to electrostatic interactions between the negatively charged sulfate groups and the positively charged amino acids of the HIV-1 gp120 protein [62]. Since HTCC is positively charged, it may experience some repulsion with the HIV-1 gp120 protein, reducing its effectiveness. These findings underscore a positive correlation with the in silico experiments.

Since HTCC did not show good activity from the studies conducted and no research has been conducted on HTCC nanoparticles against HIV-1, HTCC was nanosized to explore its potential. The nanoparticles of the HTCC derivatives showed significantly enhanced antiviral inhibition compared to the parent-modified polymers. At 12.3 μg/mL, all HTCC NPs exhibited approximately 60% inhibition against HIV-1, reaching 100% for subsequent concentrations. The IC_50_ values for HTCC-1 NPs, HTCC-2 NPs, and HTCC-3 NPs were determined to be 9.6, 11.9, and 7.9 µg/mL, respectively, as shown in Table 4. The undeniable advantage of the nanotechnology component lies in the large surface area-to-volume ratio of NPs, facilitating easier penetration into cells and ultimately achieving passive interaction with substantial internal surfaces within biological systems. In addition, the activity observed for the HTCC NPs was not due to toxicity, as only non-toxic concentrations to the target cells were used in the inhibition studies. Overall, the HTCC NPs inhibited HIV-1. Therefore, they have the potential as medicine for the inhibition of HIV-1 infection.

## 5. Conclusions

In this study, both theoretical and experimental studies were conducted to evaluate the antimicrobial activity of HTCC. In the first part of this study, the molecular docking studies revealed HTCC had a high binding affinity towards *S. aureus* protein, thus indicating it might have good antibacterial activity against *S. aureus*. However, the binding affinity towards HIV-1 protein was low, indicating that HTCC demonstrates inferior antiviral activity. In the second part of this study, three distinct derivatives of HTCC were then synthesized and characterized for their physicochemical properties. The successful synthesis of HTCC was confirmed by NMR and FTIR analyses, with XRD analysis indicating improved solubility. The in vitro evaluation showed that HTCC had antibacterial activity against *S. aureus*. However, inhibition against the HIV-1 subtype C pseudovirus revealed low activity. The in vitro results were consistent with the molecular docking results. Furthermore, nanosizing HTCC exhibited remarkable antiviral activity against HIV-1 and demonstrated increased antibacterial activity, displaying superior efficacy compared to the parent biopolymers. These findings unequivocally demonstrate that nanosizing HTCC enhances the antimicrobial efficacy of the material, emphasizing the potential of nanosized HTCC as an antimicrobial agent in diverse biomedical applications. By offering alternative approaches to pathogen control, dual-function materials have the potential to mitigate AMR, potentially reducing reliance on conventional antibiotics and antivirals.

## Figures and Tables

**Table 1:** The degree of substitution, zeta potential, and solubility of the different HTCC derivatives.

Sample	Zeta Potential SD (mV)	DQ (%)	Solubility (mg/mL)
HTCC-1	22.3 ± 1.60	31	10
HTCC-2	33.0 ± 3.51	47	35
HTCC-3	59.0 ± 1.81	58	40

**Table 2:** Zone of inhibition (mm) of the polymers against *S. aureus.*

Polymer	Inhibition Zone (mm)
	*S. aureus*
Chitosan	*
HTCC	**
HTCC NPs	**

* Inhibition zone diameter of <10 mm; ** Inhibition zone diameter between 11 and 25 mm.

**Table 3:** MIC values of the polymers and their NPs against *S. aureus.*

Polymers	MIC (μg/mL)
	*S. aureus* (ATCC 29213)
HTCC-1	313
HTCC-2	156
HTCC-3	156
HTCC-1 NPs	156
HTCC-2 NPs	78
HTCC-3 NPs	78

**Table 4:** Summary of IC_50_ of HIV-1 inhibition at the highest concentration.

Formulation	IC_50_ (μg/mL)
HTCC-1 NPs	7.9
HTCC-2 NPs	11.9
HTCC-3 NPs	9.6

## Data Availability

The raw data supporting the conclusions of this article will be made available by the authors on request.

## Supplementary Materials

The following supporting information can be downloaded at: https://www.mdpi.com/article/10.3390/polym16212999/s1, Figure S1: The structural conformation of the co-crystallized (grey) and the re-docked (green) ligand of HIV-1 gp120 protein; Figure S2: The binding site (SiteMap 1) that was used for molecular docking studies for (a) *S. aureus* (b) HIV-1; Table S1: The possible binding sites of *S. aureus,* and HIV-1 proteins detected using SiteMap tool with their properties; Figure S3: The molecular dynamics simulation of CS-HIV-1 complex at 0 ns and 200 ns; Figure S4: The color change of HTCC solution before and after titration; Figure S5: The zeta potential of the different derivatives of HTCC; Figure S6:The disk-agar diffusion method.
