# Supplementary material for: Dual Antimicrobial Activity of HTCC and Its Nanoparticles: A Synergistic Approach for Antibacterial and Antiviral Applications Through Combined In Silico and In Vitro Studies
Source: Polymers (Basel). 2024 Oct 25;16(21):2999. doi: 10.3390/polym16212999 (PMC11548688; doi:10.3390/polym16212999)
Supplement: Supplementary file 1 [file polymers-16-02999-s001.zip › polymers-3234345-supplementary.pdf]

Supporting Information

Dual Antimicrobial Activity of HTCC and its Nanoparticles: A Synergistic Approach for Antibacterial and Antiviral Applications through Combined In Silico and In Vitro Studies

Khanyisile S. Dhlamini[1,2], Cyril T. Selepe[1,2], Bathabile Ramalapa[1,3], Zamani Cele[1], Kanyane Malatji[1], Krishna K. Govender[2], Lesego Tshweu[1,3*] and Suprakas Sinha Ray[1,2*]

[1]*Centre for Nanostructures and Advanced Materials, DSI-CSIR Nanotechnology Innovation Centre, Council for Scientific and Industrial Research, Pretoria 0001, South Africa.*

[2]Department of Chemical Sciences, University of Johannesburg, Doornfontein 2028, Johannesburg, South Africa.

[3]Material Science, Innovation and Modelling (MaSIM), Faculty of Natural and Agricultural Sciences, North-West University, Mmabatho 2735, South Africa

Emails; ltshweu@csir.co. za (LT) ; rsuprakas@csir.co.za (SSR)

The Comparison Between the Co-crystallized and the Re-docked Ligand of HIV-1 gp120 Protein.

The RMSD calculated between the co-crystallized and the re-docked ligand was 1.19 Å.

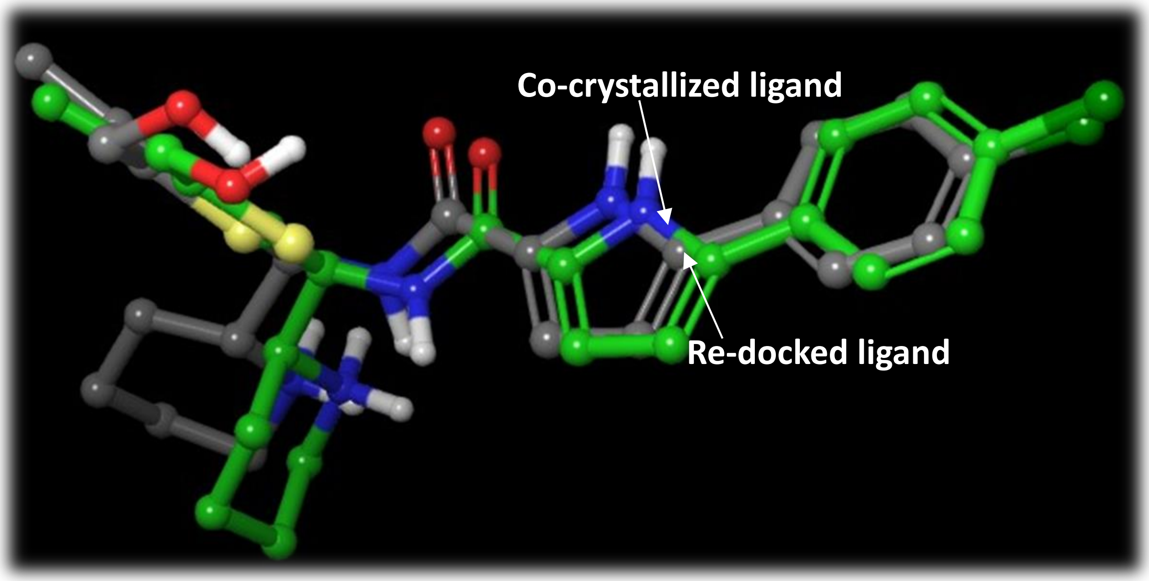

Figure S1. The structural conformation of the co-crystallized (**grey**) and the re-docked **(green**) ligand of HIV-1 gp120 protein.

Binding site detection and druggability assessment

The sitemap tool was used to analyze *S. aureus*, and HIV-1 protein structures to predict the potential binding sites and their druggability. The binding site defines the region on the protein's surface where the ligand is likely to interact. Accurate prediction of the binding site is essential for estimating the binding affinity correctly. Five binding sites were detected for *S. aureus* and HIV-1. Both *S. aureus* and HIV-1 protein had four druggable sites (SiteMap 1-4). The other sites were considered undruggable as they had a Dscore below 0.8. SiteMap 1 for *S. aureus*, and HIV-1 (**Figure S2**) were used for the molecular docking studies since they had the highest Dscore and their volume was large enough to accommodate compounds during docking

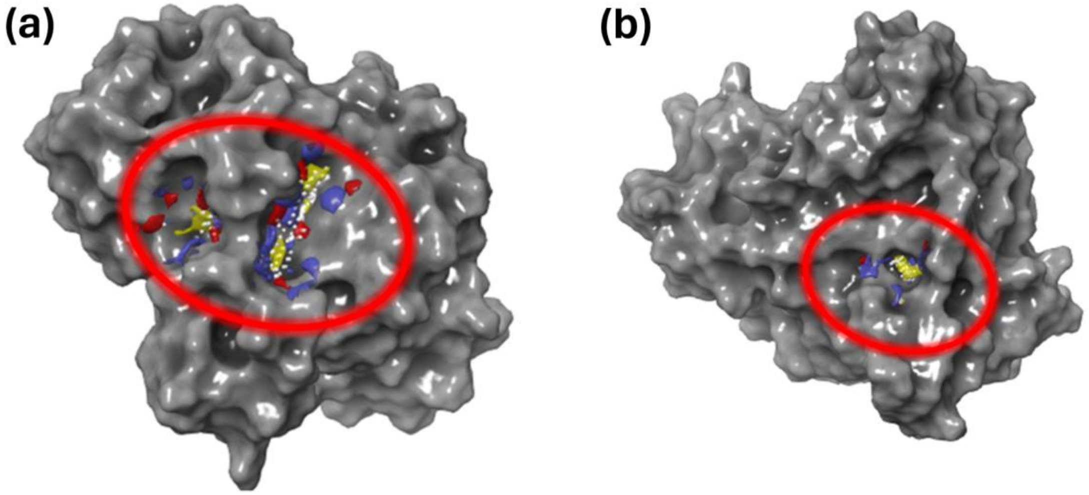

Figure S2. The binding site (SiteMap 1) that was used for molecular docking studies for a) *S. aureus* b) HIV-1

Table S1. The possible binding sites of *S. aureus,* and HIV-1 proteins detected using SiteMap tool with their properties.

**SiteMap**	***S. aureus***		**HIV-1**	
	**SiteScore**	**Dscore**	**SiteScore**	**Dscore**
**1**	**1.247**	**1.360**	**1.005**	**1.039**
**2**	**1.143**	**1.072**	**0.958**	**0.815**
**3**	**1.050**	**1.069**	**0.854**	**0.769**
**4**	**0.812**	**0.518**	**0.834**	**0.793**
**5**	**0.721**	**0.738**	**0.779**	**0.747**

Molecular Dynamics Simulations

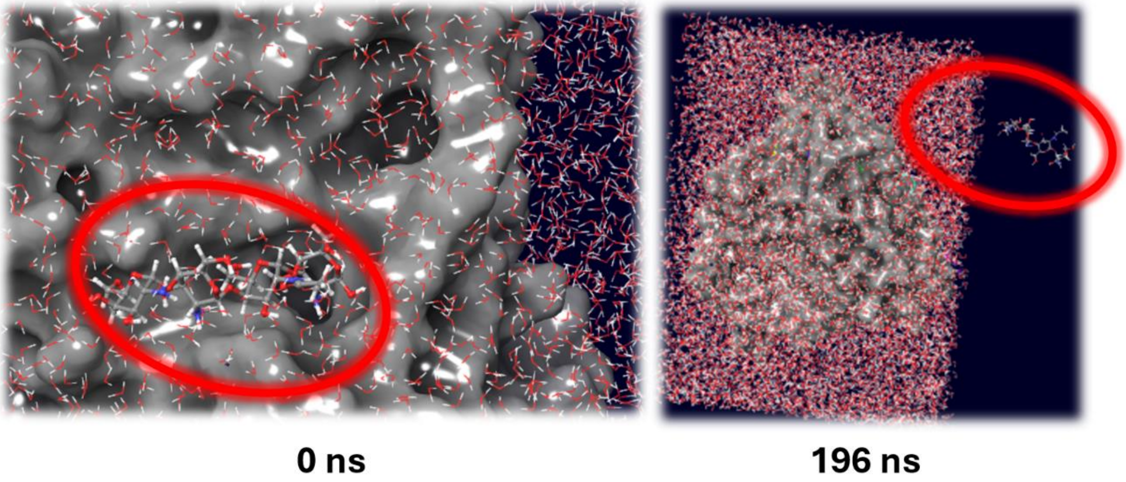

Figure S3. The molecular dynamics simulation of CS-HIV-1 complex at 0 ns and 200 ns

Determination of Degree of Quaternization. The degree of quaternization (DQ) was calculated using the titration method. The titration method was reported previously by Li *et al.*[1] HTCC (0.10 g) was dissolved in Millipore water (100 mL) and the K_2CrO_4 solution (a few drops) was added to the HTCC solution. K_2CrO_4 was used as the indicator. The HTCC-K_2CrO_4 solution was titrated with $AgNO_3$ solution (0.05M) until a red deposit formed (**Figure S4**). The DS was calculated according to the following formula:

$$DQ\ (\%) = \frac{V\ x\ c\ x\ 10^{-3}}{V\ x\ c\ x\ 10^{-3} + (W - V\ x\ c\ x\ 10^{-3}\ x\ 314)/162}\ X\ 100$$

Where V is the volume (mL) of $AgNO_3$ solution, c is the molar concentration of $AgNO_3$ and W is the mass (g) of HTCC.

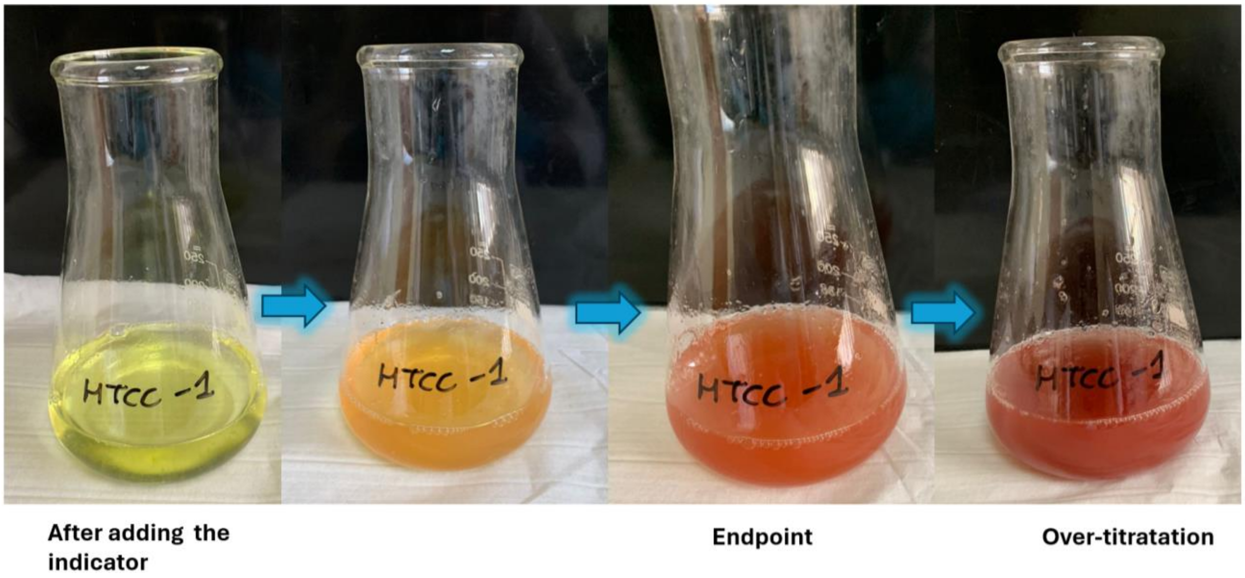

Figure S4. The color change of HTCC solution before and after titration

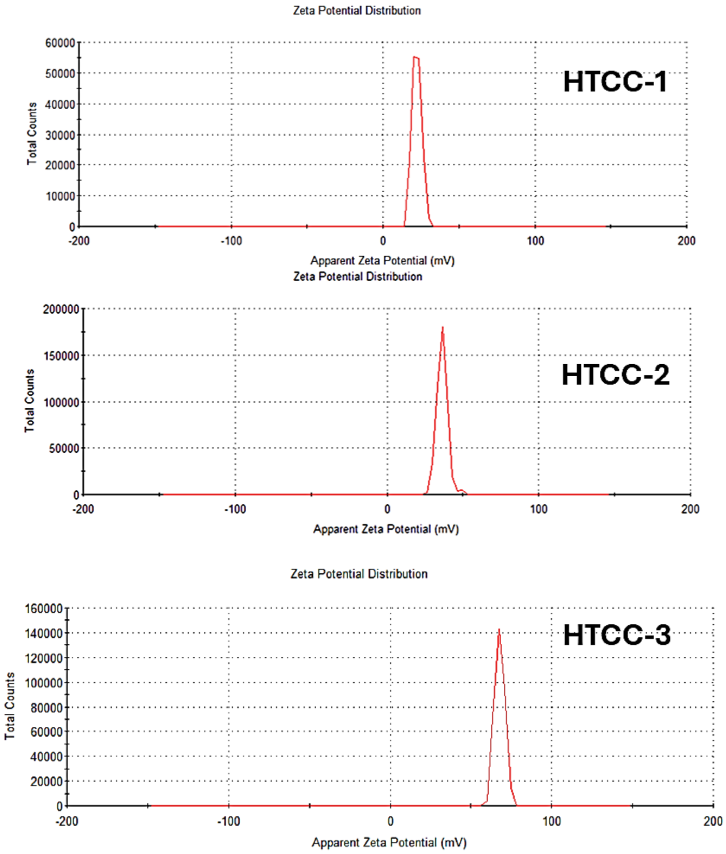

Figure S5. The zeta potential of the different derivatives of HTCC

Disk-agar Diffusion

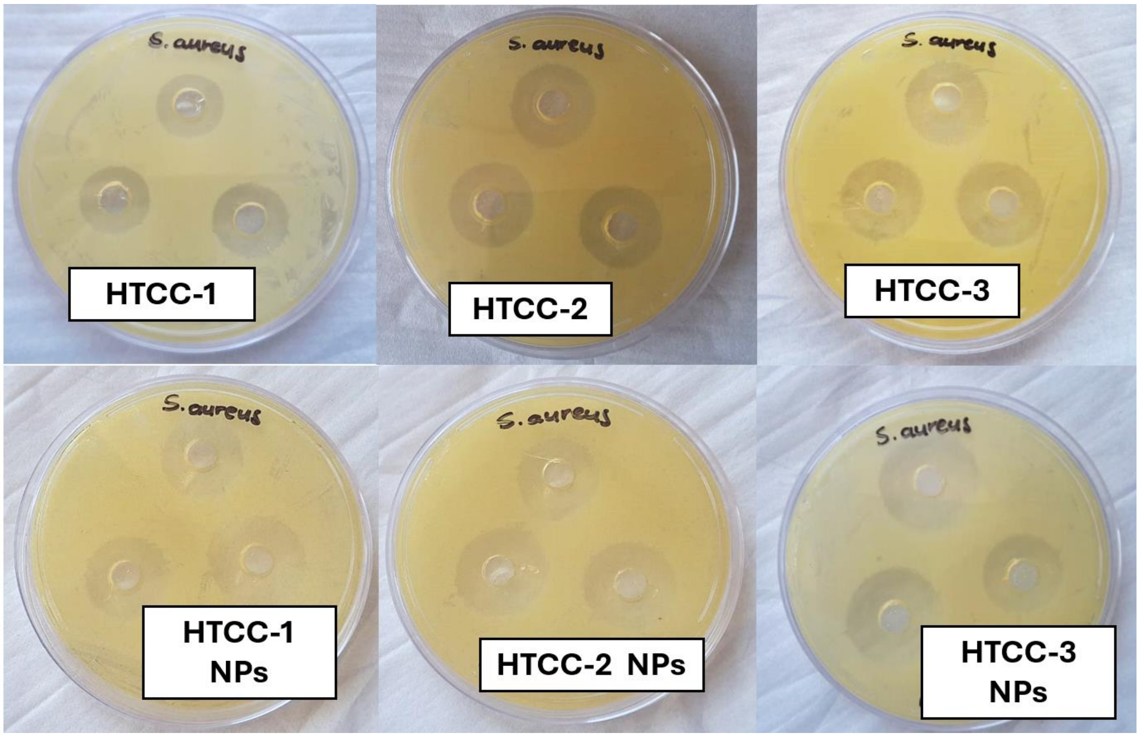

Figure S6. The disk-agar diffusion method of the different ratios of HTCCs and their NPs
